# Identification of Key Exosome Gene Signature in Mediating Coronary Heart Disease by Weighted Gene Correlation Network Analysis

**DOI:** 10.1155/2021/3440498

**Published:** 2021-10-15

**Authors:** Yanbin Fu, Yanzhi Ge, Jianfeng Cao, Zedazhong Su, Danqing Yu

**Affiliations:** ^1^School of Medicine, South China University of Technology, Guangzhou, China; ^2^The First Affiliated Hospital, Zhejiang Chinese Medical University, Hangzhou, Zhejiang, China; ^3^Department of Cardiology, Guangdong Cardiovascular Institute, Guangdong Provincial Key Laboratory of Coronary Heart Disease Prevention, Guangdong Provincial People's Hospital, Guangdong Academy of Medical Sciences, Guangzhou, China

## Abstract

**Background:**

Coronary heart disease (CHD) is the most prevalent disease with an unelucidated pathogenetic mechanism and is mediated by complex molecular interactions of exosomes. Here, we aimed to identify differentially expressed exosome genes for the disease development and prognosis of CHD.

**Method:**

Six CHD samples and 32 normal samples were downloaded from the exoRbase database to identify the candidate genes in the CHD. The differentially expressed genes (DEGs) were identified. And then, weighted gene correlation network analysis (WGCNA) was used to investigate the modules in coexpressed genes between CHD samples and normal samples. DEGs and the module of the WGCNA were intersected to obtain the most relevant exosome genes. After that, the function enrichment analyses and protein-protein interaction network (PPI) were performed for the particular module using STRING and Cytoscape software. Finally, the CIBERSORT algorithm was used to analyze the immune infiltration of exosome genes between CHD samples and normal samples.

**Result:**

We obtain a total of 715 overlapping exosome genes located at the intersection of the DEGs and key modules. The Gene Ontology enrichment of DEGs in the blue module included inflammatory response, neutrophil degranulation, and activation of CHD. In addition, protein-protein networks were constructed, and hub genes were identified, such as LYZ, CAMP, HP, ORM1, and LTF. The immune infiltration profiles varied significantly between normal controls and CHD. Finally, we found that mast cells activated and eosinophils had a positive correlation. B cell memory had a significant negative correlation with B cell naive. Besides, neutrophils and mast cells were significantly increased in CHD patients.

**Conclusion:**

The underlying mechanism may be related to neutrophil degranulation and the immune response. The hub genes and the difference in immune infiltration identified in the present study may provide new insights into the diagnostic and provide candidate targets for CHD.

## 1. Introduction

Coronary heart disease (CHD) is a collective term for disease in which the wall of the coronary arteries becomes narrowed due to fatty material accumulation [1, 2]. As the most common heart disease worldwide, it is estimated that around 200 million people suffer from CHD [3]. With the aging of society, an increasing number of CHD patients may be seen in the future. The poor prognosis of CHD seriously affects the quality of life of patients and brings a heavy burden to society [4]. Therefore, this study mainly explores the hub genes of exosomes in CHD and its regulatory function.

Exosomes are nanometer-sized vesicles (30-150 nm in diameter) secreted by most cells through exocytosis. They are encapsulated in a lipid bilayer and carry a variety of biomolecules, such as proteins, glycan, lipids, metabolites, RNA, and DNA [5]. Exosomes play a critical aspect in several pathological diseases, including cardiovascular disease [6, 7] and acute and chronic inflammation [8]. exoRBase is an exosome library derived from RNA-seq data analysis of human blood exosomes, including experimental verification from published literature [9]. It helps researchers identify molecular features in blood exosomes and triggers the discovery of new circulating biomarkers and functional implications for human diseases [10]. An important pathological feature of CHD is atherosclerosis. Exosome-mediated intercellular signaling may be a significant aspect of atherosclerotic plaque formation, affecting not only the initiation of CHD but also its progression [11]. Therefore, exosomes may be an ideal biomarker candidate and therapeutic target for CHD.

Recently, the focus has mainly shifted to screening DEGs but not exploring gene interactions [12]. Weighted gene correlation network analysis (WGCNA) is a systematic biology method and widely used to explore the connections between key modules and target disease [13, 14]. The WGCNA method generates a scale-free gene coexpression network based on Pearson's correlation matrix of genes [15]. After constructing the WGCNA network, we observed that similarly expressed genes were in the candidate module. Then, we analyzed the connection between acquired module and DEGs and finally determined the exosome genes with the most significant relation to CHD. Then, based on the genes acquired before, both functional enrichment analysis and protein-protein interaction (PPI) were performed and we revealed the potential transcriptional regulatory network in CHD, aiming to obtain new insights for CHD prevention and therapy. Besides, this is the first time in discovering the relationship between exosome genes and CHD by merged bioinformatic analysis. Therefore, the present study may advance the understanding of the underlying molecular mechanisms of CHD and may contribute to the diagnosis and treatment of CHD.

## 2. Methods

### 2.1. Search Strategy

The mRNA expression profiles, which included 12 CHD and 118 normal blood samples, were obtained from the exoRBase (http://www.exorbase.org/exoRBaseV2/download/toIndex) database [9]. The expression matrix was preprocessed using the following included criteria: (1) mRNA was filtered, and lncRNA was deleted; (2) at least one of mRNAs was nonzero in specific gene expression and (3) on the same platform. Finally, a total of 13768 mRNAs (including 6 CHD blood samples and 32 normal blood samples), which had proper expression data, were included for further analysis. Besides, the acquired genes could trace back to the GEO database (https://www.ncbi.nlm.nih.gov/geo/), and gene expression profiles of GSE100206 and GSE99985 were downloaded. Normal peripheral blood samples were collected from Shanghai Jiao Tong University School of Medicine, and CHD peripheral blood samples were collected from Fudan University Shanghai Cancer Center and Biomedical Research Institute. All the gene matrix data were performed by the single platform of Illumina HiSeq 2000 (Homo sapiens).

### 2.2. Data Preprocessing and DEG Analysis

Using the GEO database and merging two genes expressed matrix (including GSE99985 and GSE100206), the genes were preprocessed to do the next analysis. The limma (https://www.bioconductor.org/packages/release/bioc/html/limma.html) package in the R (version 4.0.5) software was used to normalize the gene expression profile of peripheral blood samples. The expression profile contained 13768 genes and was used for further study. After that, the limma package was employed to calculate DEGs. An adjusted-*P* value <0.05 and ∣log_2_ fold change (log_2_FC) | >1 were considered as a threshold.

### 2.3. WGCNA Construction

WGCNA is a system biology method used to describe the correlation patterns of genes in microarray samples and is commonly used in a variety of system biology analyses [13]. To conduct WGCNA analysis, the biochip platform (GPL11154) annotation information was used to match gene probes and gene names. The coexpression network module was constructed by the R software and the WGCNA package (https://cran.rproject.org/web/packages/WGCNA/index.html). To ensure the reliability of network construction, we first normalized the samples, then eliminated outliers, constructed a hierarchical cluster tree, and divided the genes with high and low coexpression into the same module according to their respective expression levels. Then, the adjacency matrix was transformed into a topological overlap matrix, and the corresponding dissimilarity degree was calculated. Based on hierarchical gene clustering, the modules were identified by the dynamic tree cutting method. The depth segmentation value was 2, and the minimum size cutoff value was 50. Meanwhile, the Pearson correlation matrix and adjacency matrix were to establish the information of the whole common expression network. Commonly, the value of the highest module significance was considered as the significant part.

### 2.4. Enrichment Analysis

WGCNA is a network-based method concentrating on gene sets other than individual genes, which alleviates the multiple testing problem inherent in microarray data analysis and is available for unweighted correlation networks. To evaluate its biological function, genes of the intersection between DEGs and the most significant module were selected for further functional enrichment analysis. The http://org.hs.eg/. db package (https://bioconductor.org/packages/release/data/annotation/html/org.Hs.eg.db.html) was selected to map the key genes with ensemble ID. The Clusterprofiler package (https://bioconductor.org/packages/release/bioc/html/clusterProfiler.html) was used to perform Gene Ontology (GO) functional annotations to explore and determine the potential biological function. RichPlot, colorspace, STRING, dose, and ggplot2 packages were also used as dependent packages, and three parts, including biological processes (BPs), cellular components (CCs), and molecular functions (MFs), were obtained.

### 2.5. PPI Network Construction and Identification of Hub Genes

At the protein level, the STRING database (https://string-db.org) was employed to construct a protein-protein interaction (PPI) network and then saved as a tsv file. The Cytoscape software (version 3.8.2) network analyzer was utilized to develop the interaction association of the candidate genes encoding in CHD. After that, the CytoHubba plugin was inserted and the Maximal Clique Centrality (MCC) method was calculated to find the top 10 hub genes. Besides, the MCC algorithm performs better performance in predicting hub genes in PPI networks compared with the rest of the topological algorithms. Thus, we selected the MCC algorithm to identify HCC hub genes [16].

### 2.6. Immune Infiltration Pattern

The proportion of each group of immune cells was estimated using the deconvolution method CIBERSORT (https://cibersort.stanford.edu/). We set to run mode as bulk-mode, disabled quantile normalization, and 100 permutations were set for the following significance analysis. We obtained the gene signatures to identify 22 immune cell populations (B cell naive, B cell memory, plasma cells, T cell CD8, T cell CD4 naive, T cell CD4 memory resting, T cell CD4 memory activated, T cell follicular helper, T cell regulatory (Treg), T cell gamma delta, NK cells resting, NK cells activated, monocytes, macrophage M0, macrophage M1, macrophage M2, dendritic cells resting, dendritic cells activated, mast cells resting, mast cells activated, eosinophils, and neutrophils). After filtration, the corrplot package was employed to generate a correlation heat map. The ggplot2 package was used to compare the normal group with the CHD group. Adjusted-*P* value <0.05 was considered significant to the corresponding cell type.

## 3. Results

### 3.1. Flow Diagram of the Study

**Figure 1:**
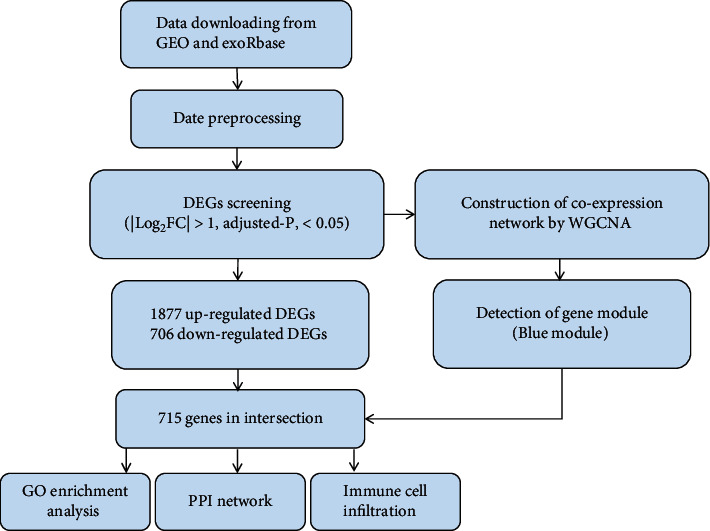
Design and workflow of the whole study. Abbreviations: DEGs: differentially expressed genes; WGCNA: weighted gene correlation network analysis; GO: Gene Ontology; PPI: protein-protein interaction.

Figure 1 shows the workflow of the study. First, the data was obtained from the exoRBase and GEO databases. After conduct batch normalization by limma package, we proceed with DEG screening and WGCNA analysis, respectively. Based on the WGCNA result, the most significant gene modules were detected. Then, taking the intersection DEGs and acquired module and the overlapping genes were regarded as the significant genes we were interested. Then, basing on DEG results, GO analyses were performed to identify the function of hub genes. The Cytoscape software was used, and 10 hub genes of the PPI network were constructed to show the interaction. Last, we analyzed the immune cell infiltrate pattern.

### 3.2. Identification of DEGs

**Figure 2:**
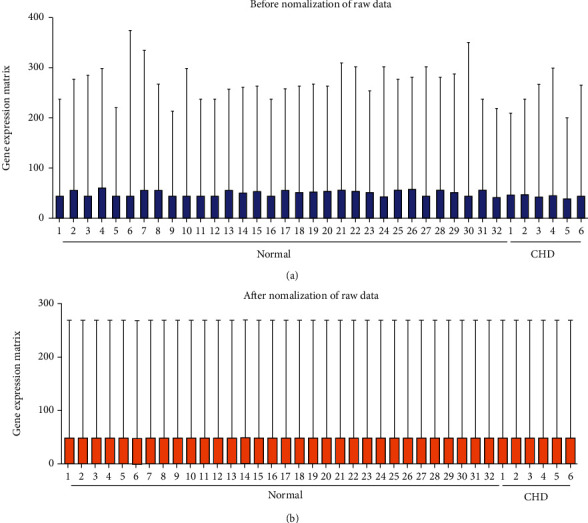
Normalization of CHD expression. (a) Expression microarray datasets of GSE100206 and GSE99985 before normalization. (b) GSE100206 and GSE99985 datasets of normalization. Abbreviations: CHD: coronary heart disease; N: normal samples.

**Figure 3:**
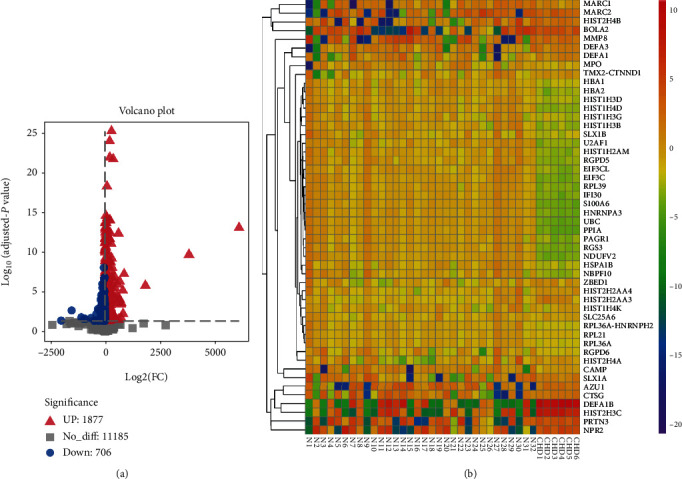
Differential analysis of datasets. (a) Differentially expressed genes screened by the criteria of ∣log_2_FC | >1 and adjusted-*P* value <0.05 and showed by volcano plot. The upregulated and downregulated genes were marked by triangles and circles, respectively. (b) The top 50 DEGs with the smallest adjusted-*P* value in the upregulated and downregulated clusters were taken out to generate a heat map, respectively. Abbreviations: DEGs: differentially expressed genes; log_2_FC: log fold change.

The GEO dataset of the blood sample was dealt with the R software. We found that there was a significant batch effect between different datasets, which was corrected by performing batch normalization in the limma package (Figure 2). Then, a total of 2583 DEGs were found. Among that, 706 genes were downregulated, and 1877 genes were upregulated in the CHD group compared with the normal group.  Figure 3(a) depicts the upregulated, downregulated genes, and non-DEGs in volcanic maps. Meanwhile, the top 50 DEGs ranked with adjusted-*P* values were used to generate a heat map (Figure 3(b)).

### 3.3. Weighted Gene Coexpression Networks and Finding Module of Interest

**Figure 4:**
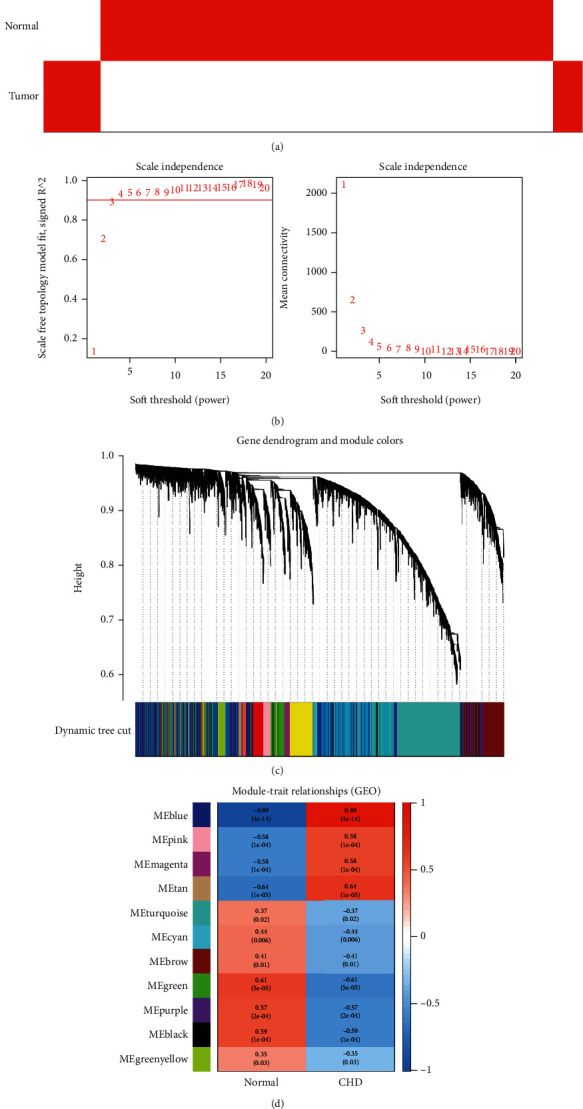
Construction of weighted gene coexpression network of CHD samples. (a) Sample clustering to detect outliers. (b) The cutoff was set to be 0.9, and *β* = 3 was chosen to be the soft-threshold power. (c) The gene dendrogram showed that the molecules were classified into different gene modules based on the correlation analysis. Different colors represented the different modules. (d) A heat map of the relationship between module traits showed the correlation between different modules and disease status. The red square represented a positive correlation, and the blue square represented a negative correlation. The common correlation between the module and the disease and the *P* value was shown in the box. (e) Correlation between module membership of blue and green circles and gene significance (absolute value). (f) Venn diagram of gene crossover between the DEG list and the blue module. A total of 715 overlapping genes were located at the intersection of the DEGs and the blue module. Abbreviation: DEGs: differentially expressed genes.

We extracted exosome sequencing genes from CHD blood samples and exosome sequencing genes from normal blood samples for WGCNA to explore coexpression networks. The CHD and normal sample cluster tree diagram is shown in Figure 4(a). We used a scale-free topology index and mean connectivity to determine the soft threshold of WGCNA. The higher the scale-free topology index value equaled to the greater the possibility of the scale-free feature. The correlation coefficient between log (*k*) and log*P* (*k*) was 0.9, and the soft threshold *β* = 3 was selected to convert the correlation matrix into a scale-free adjacency matrix (Figure 4(b)). Next, the dynamic tree cutting based on the topological overlap matrix was used to generate the coexpression module, and the coexpression network generated a total of 11 modules (Figure 4(c)). To examine the correlation between different modules and CHD conditions, we calculated the correlation factors for each module (Figure 4(d)). It showed that the correlation coefficients of the blue module, which contains 1994 genes, were greatest (Figure 4(e), correlation coefficient = 0.89, *P* < 0.001, containing 487 genes). The modules obtained from WGCNA were verified with the results of differential gene cluster analysis. To further explore the physiological or pathological pathways associated with CHD, DEGs and the blue module were intersected, and 715 overlapping genes were obtained (Figure 4(f)).

### 3.4. Functional Annotation

**Figure 5:**
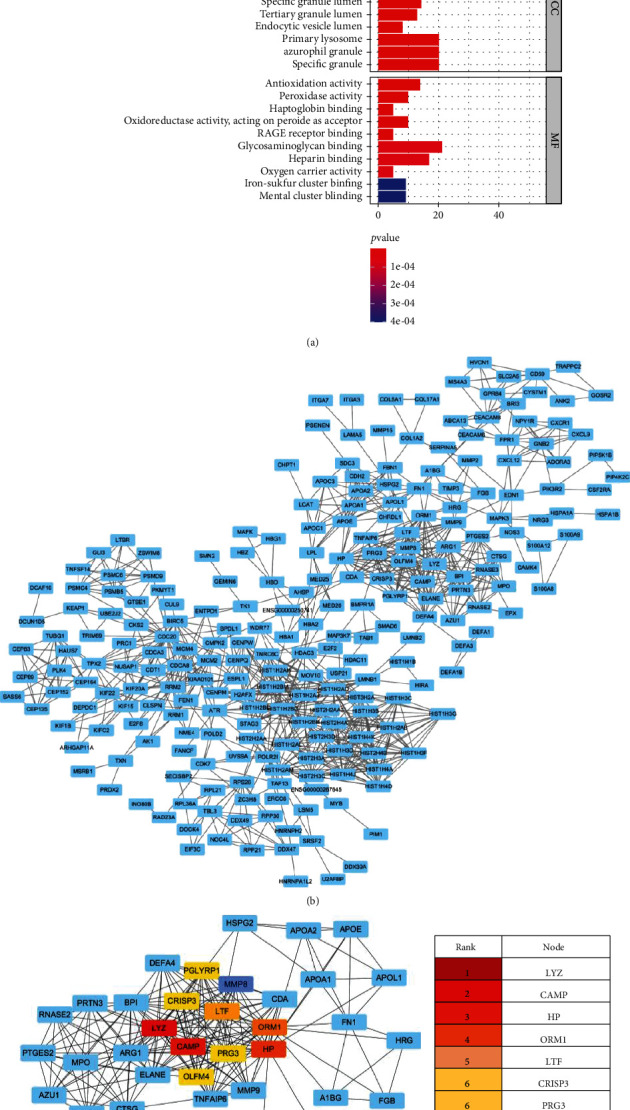
Functional enrichment analysis of DEGs. (a) GO enrichment significance items of overlapping DEGs in different functional groups: BP, CC, and MF. (b) Visualization of the PPI network and hub genes of the identified DEGs by Cytoscape of a PPI network of overlapping DEGs. (c) Ten hub genes of the overlapping DEGs marked with different colors. (d) Top 10 DEGs ranked by MCC. The redder, the higher its grades. Abbreviations: DEGs: differentially expressed genes; GO: Gene Ontology; BP: biological process; CC: cellular component; MF: molecular function; PPI: protein-protein interaction.

The GO enrichment analysis of overlapping genes is shown in Table 1 and Figure 5(a). As for BP, the analysis showed that these genes were enriched in multiple pathways, including neutrophil degranulation and activation involved in the immune response. With regard to CC, these genes were mainly involved in the formation of vesicle lumen, cytoplasmic lumen, and secretory granule lumen. And for MF, these genes were related to glycosaminoglycan binding, heparin binding, and antioxidant activity. These results suggest that CHD exosome mRNA may play an important role in regulating immune response during the occurrence and development of disease.

### 3.5. PPI Network Construction and Identification of Hub Genes

A PPI network of 715 overlapping genes was depicted using the STRING database. Hub genes were selected from the PPI network through the MCC algorithm of CytoHubba plugin (Figure 5(b)). The top 10 genes with the highest MCC scores were identified as centers and are shown in Figure 5(c), including lysozyme (LYZ), CAMP, haptoglobin (Hp), ORM1, LTF, CRISP3, PRG3, MMP8, OLFM4, and peptidoglycan recognition protein-1 (PGLYRP1) (Figure 5(d)).

### 3.6. Immune Cell Infiltration Analysis

**Figure 6:**
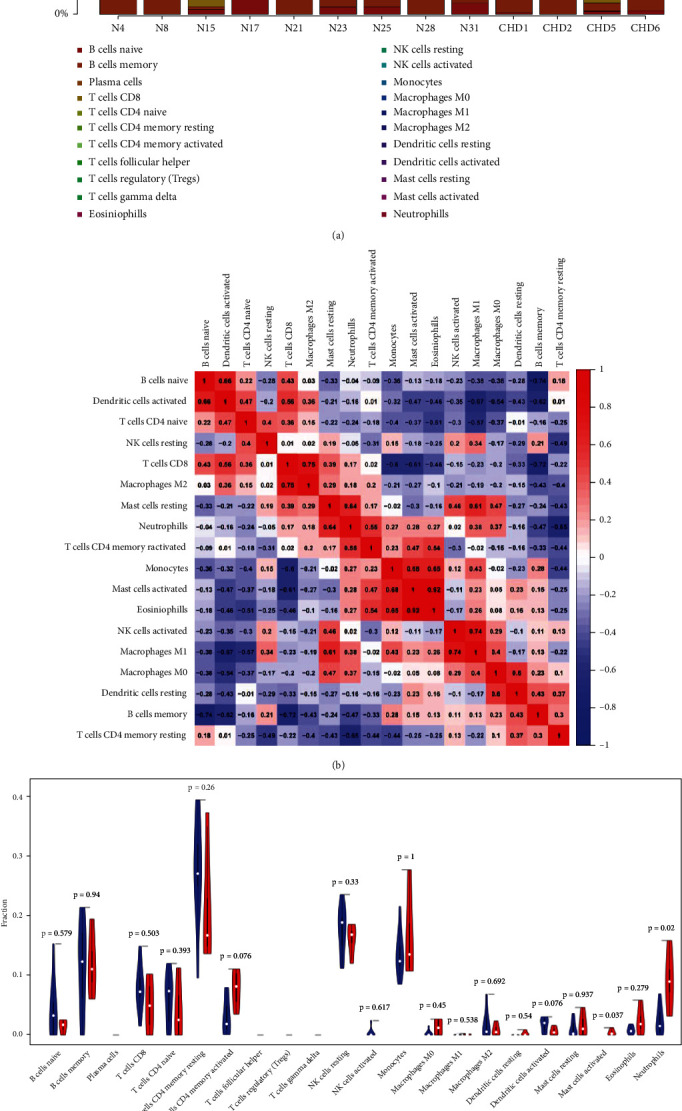
Immune cell infiltration analysis. (a) The distribution of immune infiltration in peripheral blood of CHD patients in 22 subpopulations of immune cells. Different colors represent different immune cells, and the length of the bars in the barplot represents the proportion of the immune cell population. (b) A Corheatmap of the correlation matrix for filtered 18 immune cell proportions in CHD. The red color represents the positive relationship between two immune cells, and the blue color represents the negative relationship between two immune cells. The darker the color, the higher the correlation was (*P* < 0.05). (c) The violin plot of immune cells. The blue bar represents normal samples, and the red bar represents the peripheral blood samples of CHD patients. Abbreviations: CHD: coronary heart disease; N: normal samples.

Multiple GO functional analyses related to immune processes were identified. Thus, to further discover the relationship between both, we first examined the pattern of immune infiltration under CHD and normal conditions using the CIBERSORT algorithm. The percent of the 22 immune cells is visually displayed in Figure 6(a). Corheatmap (Figure 6(b)) result showed that mast cells activated and eosinophils had a positive correlation (value = 0.92). B cell memory had a significant negative correlation (value = −0.74) with B cell naive. Besides, in CHD patients, neutrophils and mast cells were significantly increased (*P* < 0.05) and showed in the vioplot as below (Figure 6(c)).

## 4. Discussion

Exosomes had shown a critical effect on the occurrence and development of CHD. Intercellular vesicle information transport of exosomes is one of the important mechanisms of CHD [17]. The recognition and researches of exosomes were penetrating deeply and had got some new progressions for the past few years. Valadi et al. showed many mRNAs were not present in the donor cell [18]. They were passed to another cell and translated through the exosome. It had been proved the translation of exosome mRNAs was functional in vitro. To confirm the potential effects of exosome genes in the development of CHD, we screened the DEGs associated with CHD based on the exoRBase data and the most related module to obtain the candidate genes associated with CHD. In the GO enrichment analyses, most genes were enriched in BP and CC. Neutrophil degranulation, neutrophil activation involved in immune response, secretory granule lumen, cytoplasmic vehicle lumen, and vehicle lumen collagen-containing were the most remarkable categories. In this study, we used the Cytoscape software and the MCC method to further discover the core genes in the network. Then, the plugin Cytoscape was used for node ranking calculation. Chin et al. [19] have reported that CytoHubba provides 11 topological analysis methods including Degree, Edge Percolated Component, Maximum Neighborhood Component, Density of Maximum Neighborhood Component, Maximal Clique Centrality, and six centralities (Bottleneck, EcCentricity, Closeness, Radiality, Betweenness, and Stress) based on shortest paths. Among that, the newly proposed method, MCC, has a better performance on the precision of predicting essential proteins and has been adopted in this study. Finally, ten hub genes were discovered from the PPI network, including LYZ, CAMP, HP, ORM1, LTF, CRISP3, MMP8, OLFM4, and PGLYRP1.

Atherosclerosis was considered to be the pathological foundation of CHD. The development of atherosclerosis is the result of the combined action of chronic inflammation and abnormal lipid metabolism [20]. CAMP was a member of the antimicrobial peptide family with a cathelin domain characterized by a highly conserved N-terminal signal peptide [21, 22], except for its antibacterial activities, the CAMP protein functions in the inflammatory response [23], which was related to the development of CHD. Zhao et al. showed that serum levels of LL-37 (human analog of CAMP) were significantly reduced in CHD patients [24, 25]. Moreover, LL-37 was expressed in atherosclerotic lesions, mainly existed in macrophages and T cells, and functioned in inducing inflammatory gene expression [25]. Amounts of LL-37-mtDNA complex increased in atherosclerotic plasma and plaques, resisted DNase II degradation, and escaped from autophagic recognition in atherosclerosis [26], indicating that CAMP might induce atherosclerosis to increase the risk of CHD.

LYZ was a basic protein interacting with negatively charged phospholipid bilayers [27]. Endogenous LYZ regulated the composition of exosome-related RNA during inflammation, reflecting its role in cell-cell communication signaling in the inflammatory response of CHD [28]. Abey et al. demonstrated the importance of LYZ in epithelial cell migration. Meanwhile, they also found that LYZ therapy altered the expression of proteins, which was associated with signaling networks of inflammation, immune signaling, and atherosclerotic pathways [29]. Abdul-Salam et al. found that LYZ was a potential biomarker for atherosclerotic disease. The elevated LYZ level was closely correlated with disease severity, suggesting its value as a diagnostic tool to assess CHD patients [30].

PGLYRP1 was a bacterial wall component known to be present in human atherosclerosis and was found almost exclusively in the secretory granules of neutrophils and eosinophils [31]. Peptidoglycan might promote inflammation by activating innate immune responses, peptidoglycan recognition proteins, chemokines, and proinflammatory cytokines (IL-1, IL-6, and tumor necrosis factor-alpha) in nonmucosal sites [32]. These processes may promote and accelerate the development of atherosclerotic lesions. Our results verified that CAMP, LYZ, and PGLYRP1 were important factors in constituting the atherosclerosis of CHD.

The role of cholesterol in coronary heart disease was undisputed [33, 34]. Hp was a rich plasma protein that played an important role in immune regulation and reversal of cholesterol transport. It is also bound to hemoglobin to protect against oxidative damage [35]. Also, this gene played an important role in CHD pathological process [36, 37]. In Belgian, Hp 1-1 phenotype had a strong association with an increased risk of CHD [38]. It had been reported by Cahill et al. that diabetes mellitus individuals carried with Hp 2-2 allele had more likely to develop CHD [39], which means that Hp was closely related to the occurrence and development of cardiovascular disease.

Other key exosome genes, including LTF, CRISP3, and OLFM4, were mainly associated with acute and chronic inflammation. MMP8, LTF, CRISP3, and OLFM4 were upregulated in the inflammatory process to facilitate leukocyte-mediated migration, neutrophil activation, and degranulation process [40]. MMP8, LTF, and OLFM4 were also known as neutrophil collagenase [41]. MMP8 was expressed and produced by endothelial cells, smooth muscle cells, and macrophages in atherosclerotic plaques [42]. Momiyama et al. proved MMP8 levels were higher in both stable CHD and unstable angina patients. Also, high plasma MMP8 levels suggested that MMP8 might reflect coronary plaque instability, which suggested that MMP8 was a promising biomarker for CHD [43]. OLFM4 and LTF were subpopulations of neutrophils in septic shock. Among that, a high percentage of OLFM4 positive neutrophils were associated with a greater risk of organ failure and death [44]. PRG3 gene was a novel p53 target gene in p53-dependent apoptosis pathway [45].

Infiltrated immune cells constitute important parts of CHD and have been widely studied in recent years. Neutrophil was found to be indicative of responses to plaque formation [46]. It expelled intracellular contents which were rich in uncoagulated chromatin, histones, and active substances [47]. These intracellular contents could participate in plaque erosion, including noxious effects on vascular cells, direct thrombogenic activity, and the promotion of platelet activation/aggregation [47]. Quillard et al. had demonstrated that TLR2 stimulation and neutrophil participation might cause the plaques vulnerable to superficial erosion and thrombotic complications [48]. Therefore, the function of neutrophils played a critical role in the pathophysiology of CHD, which is consistent with our results.

Some limitations cannot be ignored. First of all, the results in this paper were limited to bioinformatic analysis, and it had not been further proved by experiments. Second, the number of samples was limited because of insufficient databases. Therefore, further verification was needed by collecting more clinical samples. Moreover, studies were needed to explore how these exosome genes work in vivo and in vitro.

In summary, we determined that the activation and degranulation of neutrophils may possess significant roles in mediating the process of CHD. Besides, the underlying mechanism may be related to the immune cell infiltration response. In addition, the core PPI exosome genes identified might be used as biomarkers and therapeutic targets for CHD. This study could provide a new insight to predict, assess, and treat for CHD.

## Figures and Tables

**Table 1:** GO analysis of overlapping genes.

Category	ID	Description	Adjust*-P* value	Genes
BP	GO:0043312	Neutrophil degranulation	3.25*E*-11	DEFA1B/HSPA1B/CAMP/MPO/AZU1/MMP8/DEFA1/CTSG/PRTN3/PGAM1/MMP9/ELANE/S100A8/HP/EPX/LTF/PRG2/PGLYRP1/CEACAM6/S100A9/CYSTM1/CDA/LYZ/ORM1/FCGR3B/DEFA4/S100A12/BPI/OLFM4/PRG3/LGALS3/RNASE2/HVCN1/A1BG/MS4A3/SLC2A5/ABCA13/HSPA1A/S100P/TNFAIP6/CD59/GPR84/FPR1/ARG1/RNASE3/BRI3/CXCR1/CRISP3/CEACAM8/PRAM1/MME/ALAD/PTGES2
BP	GO:0002283	Neutrophil activation involved in immune response	3.25*E*-11	DEFA1B/HSPA1B/CAMP/MPO/AZU1/MMP8/DEFA1/CTSG/PRTN3/PGAM1/MMP9/ELANE/S100A8/HP/EPX/LTF/PRG2/PGLYRP1/CEACAM6/S100A9/CYSTM1/CDA/LYZ/ORM1/FCGR3B/DEFA4/S100A12/BPI/OLFM4/PRG3/LGALS3/RNASE2/HVCN1/A1BG/MS4A3/SLC2A5/ABCA13/HSPA1A/S100P/TNFAIP6/CD59/GPR84/FPR1/ARG1/RNASE3/BRI3/CXCR1/CRISP3/CEACAM8/PRAM1/MME/ALAD/PTGES2
BP	GO:0050832	Defense response to fungus	4.58*E*-07	DEFA1B/DEFA3/MPO/DEFA1/CTSG/ELANE/S100A8/LTF/S100A9/DEFA4/S100A12/HRG/ARG1
BP	GO:0019730	Antimicrobial humoral response	6.57*E*-07	DEFA1B/CAMP/DEFA3/AZU1/DEFA1/CTSG/PRTN3/ELANE/S100A8/LTF/PGLYRP1/S100A9/LYZ/DEFA4/S100A12/BPI/CXCL9/HRG/BCL3/PGLYRP2/FGB/RNASE3
BP	GO:0031640	Killing of cells of other organism	1.68*E*-06	DEFA1B/CAMP/DEFA3/AZU1/DEFA1/CTSG/ELANE/LTF/PGLYRP1/LYZ/DEFA4/S100A12/HRG/ARG1/APOL1
CC	GO:0034774	Secretory granule lumen	1.77*E*-09	DEFA1B/CAMP/DEFA3/MPO/AZU1/MMP8/DEFA1/CTSG/PRTN3/PGAM1/ELANE/S100A8/HP/EPX/LTF/PGLYRP1/S100A9/CDA/LYZ/ORM1/FN1/DEFA4/S100A12/TIMP3/BPI/OLFM4/PRG3/RNASE2/A1BG/HRG/S100P/FGB/ARG1/RNASE3/APOA1/CRISP3/ALAD/PTGES2
CC	GO:0060205	Cytoplasmic vesicle lumen	1.77*E*-09	DEFA1B/CAMP/DEFA3/MPO/AZU1/MMP8/DEFA1/CTSG/PRTN3/PGAM1/ELANE/S100A8/HP/EPX/LTF/PGLYRP1/S100A9/CDA/LYZ/ORM1/FN1/DEFA4/S100A12/TIMP3/BPI/OLFM4/PRG3/RNASE2/A1BG/HRG/S100P/FGB/ARG1/RNASE3/APOA1/CRISP3/ALAD/PTGES2
CC	GO:0031983	Vesicle lumen	1.77*E*-09	DEFA1B/CAMP/DEFA3/MPO/AZU1/MMP8/DEFA1/CTSG/PRTN3/PGAM1/ELANE/S100A8/HP/EPX/LTF/PGLYRP1/S100A9/CDA/LYZ/ORM1/FN1/DEFA4/S100A12/TIMP3/BPI/OLFM4/PRG3/RNASE2/A1BG/HRG/S100P/FGB/ARG1/RNASE3/APOA1/CRISP3/ALAD/PTGES2
MF	GO:0016209	Antioxidant activity	0.000793131	HBA1/MPO/HBA2/HP/EPX/S100A9/PRDX2/APOE/TXN/HBM/HBZ/TXNDC17/HBG1/PXDNL
MF	GO:0004601	Peroxidase activity	0.002388549	HBA1/MPO/HBA2/EPX/PRDX2/HBM/HBZ/TXNDC17/HBG1/PXDNL
MF	GO:0031720	Haptoglobin binding	0.002388549	HBA1/HBA2/HBM/HBZ/HBG1

Abbreviations: BP: biological process; CC: cellular component; MF: molecular function.

## Data Availability

All data can be obtained from the corresponding author Danqing Yu or the first author Yanbin Fu.
